# 2102. Common co-occurence in the respiratory tract of Mucorales with Gram-negative rods in patients with hematologic malignancy and sinopulmonary mucormycosis

**DOI:** 10.1093/ofid/ofac492.1724

**Published:** 2022-12-15

**Authors:** Stephanie L Egge, Sebastian Wurster, Dierdre B Axell-House, Ying Jiang, William R Miller, Dimiitrios P Kontoyiannis

**Affiliations:** Houston Methodist Hospital/Houston Methodist Research Institute, Houston, Texas; The University of Texas MD Anderson Cancer Center, Houston, Texas; Houston Methodist Research Institute, Houston, Texas; The University of Texas MD Anderson Cancer Center, Houston, Texas; Houston Methodist Hospital and Weill Cornell Medical College, Houston, Texas; The University of Texas MD Anderson Cancer Center, Houston, Texas

## Abstract

**Background:**

Mucorales (MCR) and Gram-negative rods (GNR) commonly infect patients (pts) with hematological malignancies (HM); however, their co-occurrence is understudied.

**Methods:**

We retrospectively reviewed the records of 63 pts with HM and sinopulmonary MCR (proven or probable, EORTC/MSG criteria) at MD Anderson Cancer Center (Houston, TX) from 2008-2016. Co-occurrence was defined as a culture positive for GNR taken from sinuses or the lungs within 90 days (d) of a positive Mucorales culture or histology demonstrating Mucorales spp.

**Results:**

Ninety percent of the 63 pts with MCR had leukemia or myelodysplastic syndrome (54% AML) and 10% had lymphoma/myeloma. Sixty-eight percent had undergone hematopoietic stem cell transplant. Sixty-seven percent were neutropenic at the time of MCR diagnosis. Thirty-five percent had received > 600mg of prednisone within 30 days (d) prior to MCR diagnosis. Thirty-five percent were admitted to the ICU during the time of MCR treatment. MCR pts had sinusitis (42.9%), pneumonia (38.1%) and disseminated sinopulmonary disease (19.0%). Nearly all pts (92%) received empiric antimicrobials with activity against Pseudomonas prior to collection of their positive fungal cultures. Twenty-three pts (37%) had concurrent detection of GNRs (Pseudomonas in 10, Stenotrophomonas in 8) in samples from the sinopulmonary tract. Eight of 23 co-isolations of GNRs and Mucorales (35%) were found in same-day samples. Demographic and clinical characteristics of pts with and without co-occurrence of GNR were comparable. Pts with co-occurrence had less frequently received antibiotics with activity against Stenotrophomonas 7 (30.4%) vs 23 (60%, p=0.024). Ninety-day all-cause mortality was high and comparable in pts with (83%) and without (78%) GNR detection (p = 0.75).

**Conclusion:**

Over a third of heavily immunosuppressed pts with sinopulmonary MCR harbor GNRs in their respiratory tract, most commonly Pseudomonas. Although no impact on survival outcomes was seen in a background of high mortality, pathogenesis studies are needed to understand the mutualistic interplay of GNR and Mucorales spp. and their influence on host responses.

**Disclosures:**

**William R. Miller, MD**, Entasis Therapeutics: Grant/Research Support|UpToDate: Royalties - Topic Contributor **Dimiitrios P. Kontoyiannis, MD, ScD, PhD (hon)**, AbbVie: Advisor/Consultant|Astellas Pharma: Advisor/Consultant|Astellas Pharma: Grant/Research Support|Astellas Pharma: Honoraria|Cidara Therapeutics: Advisor/Consultant|Gilead Sciences: Advisor/Consultant|Gilead Sciences: Grant/Research Support|Gilead Sciences: Honoraria|Merck: Advisor/Consultant.

